# Development of the simultaneous determination of anionic and cationic nutrients in hydroponic solutions using polymer-based zwitterionic exchanger with neutral eluent

**DOI:** 10.1007/s44211-026-00922-0

**Published:** 2026-05-19

**Authors:** Yuki Uga, Atsushi Hashigami, Haruki Tsuboi, Yamato Okada, Yuta Mitsui, Taku Fujiwara, Yuki Sago, Daisuke Kozaki

**Affiliations:** 1 Department of Chemistry and Biotechnology, Faculty of Science and Technology, Kochi University, 2-5-1 Akebono-cho, Kochi City, Kochi, 780-8520 Japan; 2 Department of Environmental Engineering, Kyoto University, C1-222, Nishikyo-ku, Kyoto, 615-8540 Japan; 3 Graduate School of Sciences and Technology for Innovation, Yamaguchi University, 1677-1, Yoshida, Yamaguchi, 753-8515 Japan

**Keywords:** Zwitterionic exchanger (sulfobetaine group), Neutral eluent, Ion-exclusion/cation-exchange chromatography, Ionic nutrient, Hydroponic culture

## Abstract

In hydroponic cultivation, pH and electrical conductivity are currently widely used as indicators for nutrient management, whereas the concentrations and compositions of individual ions (NO_3_^‒^, NO_2_^‒^, NH_4_^+^, H_2_PO_4_^‒^/HPO_4_^2‒^, K^+^) are not precisely monitored. To reduce the environmental load and nutrient costs in future hydroponics, individual fertilization management based on effective and detailed monitoring of nutrients is required, necessitating accurate evaluation of nutrient levels. In this study, we describe the development and application of ion exclusion/cation exchange chromatography (IEC/CEC) with a neutral eluent (40 mM sodium acetate, and 0.5 mM 18-crown-6, pH 7.71) for simultaneous separation of ionic nutrients in a hydroponic fertilizer solution. These problems of the degradation of the separation column due to the precipitation of metal ion species under alkaline elution condition and the oxidation of NO_2_^‒^ to NO_3_^‒^ encountered under acidic eluent used in previously reported chromatographic studies were avoided while ensuring the base gel stability by using a polymer-based zwitterionic exchanger with neutral eluent. In addition, the developed IEC/CEC method was successfully applied to monitor the temporal evolution of the amount of target ionic nutrients in hydroponic solutions.

**Graphical abstract:**

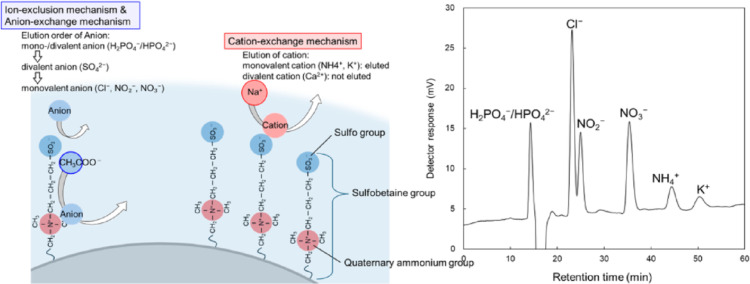

**Supplementary Information:**

The online version contains supplementary material available at 10.1007/s44211-026-00922-0.

## Introduction

Various analytical methods such as atomic absorption spectroscopy, electrically modulated chromatography, liquid chromatography inductively coupled plasma mass spectrometry, capillary electrophoresis, and ion chromatography (IC) have been developed for quantification of inorganic ions [1–7]. Above mentioned analytical methods, IC offers high separation efficiency and sensitivity, making it suitable for analysis of ionic substances [5, 6]. In addition, the excellent selectivity, sensitivity, and reproducibility of IC make it an ideal technique for analysis of trace inorganic ions in various samples. In IC separation, anion-exchange chromatography (AEC), cation-exchange chromatography (CEC), and ion-exclusion chromatography (IEC) are dominantly used. Generally, basic eluent is used for AEC while acidic eluent is used for the CEC, and IEC [5]. In addition, mix-mode chromatography (MMC) is becoming increasingly popular for the simultaneous separation of two or more different target analytes such as organic, and inorganic anions, and cations [5, 8–12]. Moreover, MMC allows simultaneous quantification of multiple ions in various complex samples, enabling rapid and accurate evaluation of various fields of water quality monitoring, and management.

We investigated the application of MMC for simultaneous analysis of anionic and cationic species in nutrient solutions used in hydroponic cultivation, and the results are presented in Table 1 [13–15]. Many hydroponic facilities use pH and electrical conductivity (EC) as indicators for nutrient management, whereas the concentrations of individual ions (NO_3_^‒^, NO_2_^‒^, NH_4_^+^, H_2_PO_4_^‒^/HPO_4_^2‒^, K^+^) are not precisely monitored [16–20]. The optimization of fertilizer use is especially important given the recent surge in fertilizer market prices [21]. In addition, given that spent nutrient solutions comprising high concentrations of various nutrients are eventually discharged, their composition should be appropriately managed to protect the water quality in the receiving natural environment [22–25].

**Table 1:** Details (eluent, separation column, separation mode, analytes, and problems) of the previously reported analytical methods for the monitoring of ionic nutrients

Eluent (pH)	Separation column (exchange groups and column packing material)	Separation mode	Analytes	Problems	Reference
3.5 mM NaOH (pH 11.57)	TSKgel Super IC-AZ (150 mm × 4.6 mm i.d.) Exchange group: Quaternary ammonium group Base material: Hydrophilic porous polymer	CEC/IEC	NO_3_^‒^, NO_2_^‒^, HPO_4_^2‒^/H_2_PO_4_^‒^, Cl^‒^, HCO_3_^‒^, NH_4_^+^, K^+^, Mg^2+^, Ca^2+^	Precipitation of Mg^2^⁺, Ca^2^⁺, and other metal species as hydroxides by the alkaline eluent, leading to column degradation	[15]
5 mM L-tartaric acid and 3 mM 18-crown-6 (pH 2.98)	TSKgel SuperIC-A/C (150 mm × 6.0 mm i.d.) Exchange group: Carboxy group Base material: Polymethacrylate Shodex SH-1011 (300 mm × 8.0 mm i.d.) Exchange group: Sulfo group Base material: Styrene–divinylbenzene copolymer	IEC/CEC	NO_3_^‒^, HPO_4_^2‒^/H_2_PO_4_^‒^, Cl^‒^, SO_4_^2‒^, NH_4_^+^, K^+^, Mg^2+^, Ca^2+^	Oxidization of NO₂^‒^ to NO₃⁻ under acidic elution conditions	[13]
20 mM HCOONa (pH 7.87)	Acclaim Trinity P2 (50 mm × 3.0 mm i.d.) Exchange group: Weakly acidic cation exchangers (inner-pore area of silica), strongly basic anion exchangers (nanopolymer particles) Base material: High-purity porous (pore size: 300 Å) spherical silica particles coated with nanopolymer particles (1000–3000 Å)	CEC/AEC	NO_3_^‒^, NO_2_^‒^, HPO_4_^2‒^/H_2_PO_4_^‒^, Cl^‒^, NH_4_^+^, K^+^	Substitution with organic solvents required after each use for preventing the degradation of the silica material	[14]

In previous our research, while ion-exchange-mode IC with basic eluents achieved determination of the anionic and cationic nutrients in fertilizer solution, it led to the precipitation of Mg^2+^, Ca^2+^, and other metal species in nutrient solutions as hydroxides, leading to moderate column degradation [15]. Ion-exclusion- and cation-exchange chromatography (IEC/CEC) with acidic eluent achieved simultaneous separation of anionic and cationic nutrients in fertilizer solution but could not achieve the determination of NO_2_^‒^ because it was oxidized to NO_3_^‒^ under acidic elution conditions [13]. By contrast, cation- and anion-exchange chromatography using a silica-based ion exchanger with neutral eluent achieved the simultaneous separation of anionic and cationic nutrients without either precipitation of metal species or oxidation of NO_2_^‒^ to NO_3_^‒^ [14]. However, the aqueous neutral eluent caused moderate degradation of the silica column unless it was substituted with organic solvents such as acetonitrile after each use. This method is totally impractical for applying to the agricultural facilities.

Despite advances in the analysis of ionic nutrients, the above-mentioned IC systems still do not satisfy all requirements for their effective application in hydroponics, as indicated in Table 1. Therefore, in the present study, we developed an IC method using the SeQuant ZIC-*p*HILIC polymer-based column, which possesses zwitterionic functional groups derived from sulfo and quaternary ammonium groups, in combination with a mixed eluent of sodium acetate and 18-crown-6. The novelty of this study is that the proposed system achieved sufficient separation of the target ions based on both the anion exclusion and cation exchange mechanisms using a neutral eluent, while resolving all the limitations mentioned earlier (Table 1). In addition, the neutral solution used as eluent is unique condition compared with the previous studied for the simultaneous separation of anions, and cations. These advances allow long-term and reproducible analysis in practical use and pave the way for applying this system as a practical monitoring alternative to pH or EC meters in agricultural facilities.

## Experimental

### Instrumentation

The developed IC system consists of a dual-head plunger pump for the eluent (LC-10AD, Shimadzu Corporation, Kyoto, Japan), a column oven for separation (CTO-10A, Shimadzu Corporation), an autosampler with a cooler maintained at 3 °C (SIL-10AF, Shimadzu Corporation), and a conductivity detector (CDD-6A, Shimadzu Corporation). Instrument control was performed using a Chromato-PRO (Runtime Instruments, Tokyo, Japan). The eluent flow rate was set to 0.25 mL/min, and the column temperature was maintained at 4 °C to minimize backpressure.

### Separation column

Three commercially available SeQuant ZIC-*p*HILIC column packed with polymer-based zwitterionic exchanger (150 mm × 4.6 mm i.d., particle size: 5 μm, Merck, Darmstadt, Germany) was used in this study. The zwitterionic exchanger of this column is a sulfobetaine group containing a terminal sulfo group (–SO_3_^‒^), and a quaternary ammonium group (–NR_3_^+^) on the stationary-phase side. These zwitterionic functional groups are chemically bound to the polymer-based gel, providing a dual mode that enhances the selectivity for a wide range of analytes based on hydrophilic and electrostatic interactions.

### Reagents

Guaranteed reagent or Wako special-grade reagents were purchased from Fujifilm Wako Pure Chemical Corp. (Osaka, Japan). Ultra-pure water (> 18 M cm, Purelab Quest 2, Elga Veolia, High Wycombe, UK) was used to prepare the standard solutions and eluents. Sample and eluent stock solutions of NH_4_NO_3_, KH_2_PO_4_, NaNO_2_, CaCl_2_, MgSO_4_, sodium acetate, and 18-crown-6 were prepared by dissolving appropriate quantities of each solute at a concentration of 0.1 M. The stock solutions were diluted with appropriate volumes of water to prepare working solutions.

### Analytical methods used for validation

Owing to the lack of official regulated analytical method for the monitoring of ionic species in hydroponic nutrient solutions, the ion concentrations except NO_2_^‒^ obtained in this study were validated by comparison with the IEC/CEC with acidic eluent [13]. The IEC/CEC with acidic eluent was performed using two Tosoh TSKgel Super-IC-A/C (150 mm × 6.0 mm i.d.), and a Shodex SH-1011 (300 mm × 8.0 mm i.d.) connected in tandem with a mixture of 5 mM L-tartaric acid, and 3 mM 18-crown-6 as the eluent. The flow rate was 1.0 mL/min, and the column temperature was 55 °C.

In addition, NO_2_^‒^ concentration obtained in this study was validated by comparison with the IC with basic eluent [15]. The IC with basic eluent measurement was performed using two Tosoh TSKgel Super-IC-AZ (150 mm × 4.6 mm i.d.) with a 3.5 mM NaOH as the eluent. The flow rate was 0.6 mL/min, and the column temperature was 40 °C.

### Noncirculating hydroponic system and other components

This study used a closed hydroponic system (Green Farm UH- A01E1) purchased from Uing (Osaka, Japan) equipped with LED lights, a pump for bubbling, and a water-level float (maximum water volume of system: 4.0 L, width: 544 mm, depth: 262 mm, height: 305 mm, weight: 5.0 kg, seeding panel: 300 g, fertilizer solution storage: 560 g).

Fertilizer powders (OAT House S1 and 2) were purchased from OAT Agrio Co., Ltd. (Tokyo, Japan). Fertilizer powder House S1 contains NO_3_^‒^–N: 8.6%, P_2_O_5_: 7.0%, K_2_O: 32.0%, MgO: 4.0%, MnO: 0.05%, B_2_O_3_: 0.07%, Fe: 0.15%, Cu: 0.002%, Zn: 0.006%, and Mo: 0.002%, while House 2 contains NO_3_^‒^–N: 11.0% and CaO: 0.07%. Liquid fertilizers were prepared by dissolving OAT House S1 and 2 fertilizer powders in ultrapure water at the concentrations of 1.5 and 1.0 g/L, respectively. Lettuce (*Lactuca sativa*, Frillice®, Snow Brand Seed Co., Hokkaido, Japan), which is widely cultivated in greenhouses and closed hydroponic horticulture, was used as a model crop.

### Hydroponic cultivation processes

Twelve lettuce seeds were seeded during the hydroponic cultivation. The hydroponic cultivation system used in this study is shown in Fig. S1 (weight of the hydroponic cultivation system = 5.0 kg [seeding panel = 300 g and fertilizer solution storage = 560 g]). During the cultivation program, the lighting system was turned off for 72 h after seeding. After 72 h, the lighting system was turned on from 06:00 to 22:00 and turned off from 22:00 to 06:00. Throughout the hydroponic cultivation, the temperature was controlled at 23–24 °C by a room air-conditioning system. The aeration system was turned on for 5 min every hour from 06:00 to 22:00.

The method for measuring the fertilizer solution volume and plant (Lactuca sativa) weight are as follows. The weight of the plant is equal to the total weight of the seeding panel, lettuce, fertilizer solution storage, and fertilizer solution (Fig. S1c) minus the total weight of the fertilizer solution storage (560 g), fertilizer solution (Fig. S1d), and the seeding panel (Fig. S1e: 300 g). In addition, the weight of the fertilizer solution is equal to the total weight of the fertilizer solution storage (560 g) and fertilizer solution (Fig. S1d) minus the weight of the fertilizer solution storage (560 g).

### Sample collection, treatment, and measurement

During the sample collection phase, fertilizer solution samples (5.0 mL) were collected on days 0, 3, 6, 9, 12 and days 15–25. After passing through a 0.45-μm polytetrafluoroethylene filter vial (Captiva®, Agilent Technologies, Inc, CA, USA), they were immediately injected into the IEC/AEC system. The excess fertilizer solution samples were refrigerated at 5 °C.

## Results and discussion

### Optimization of sodium acetate concentration in the eluent for separation of ionic nutrients

In this study, sodium acetate was selected as the eluent for the following three reasons: (1) acetate and sodium ions are not target analytes, (2) the retention times of sodium acetate do not overlap with those of the analytes, and (3) the theoretical pH of sodium acetate solutions falls within the neutral range (pH 7.7–9.2).

**Fig. 1:**
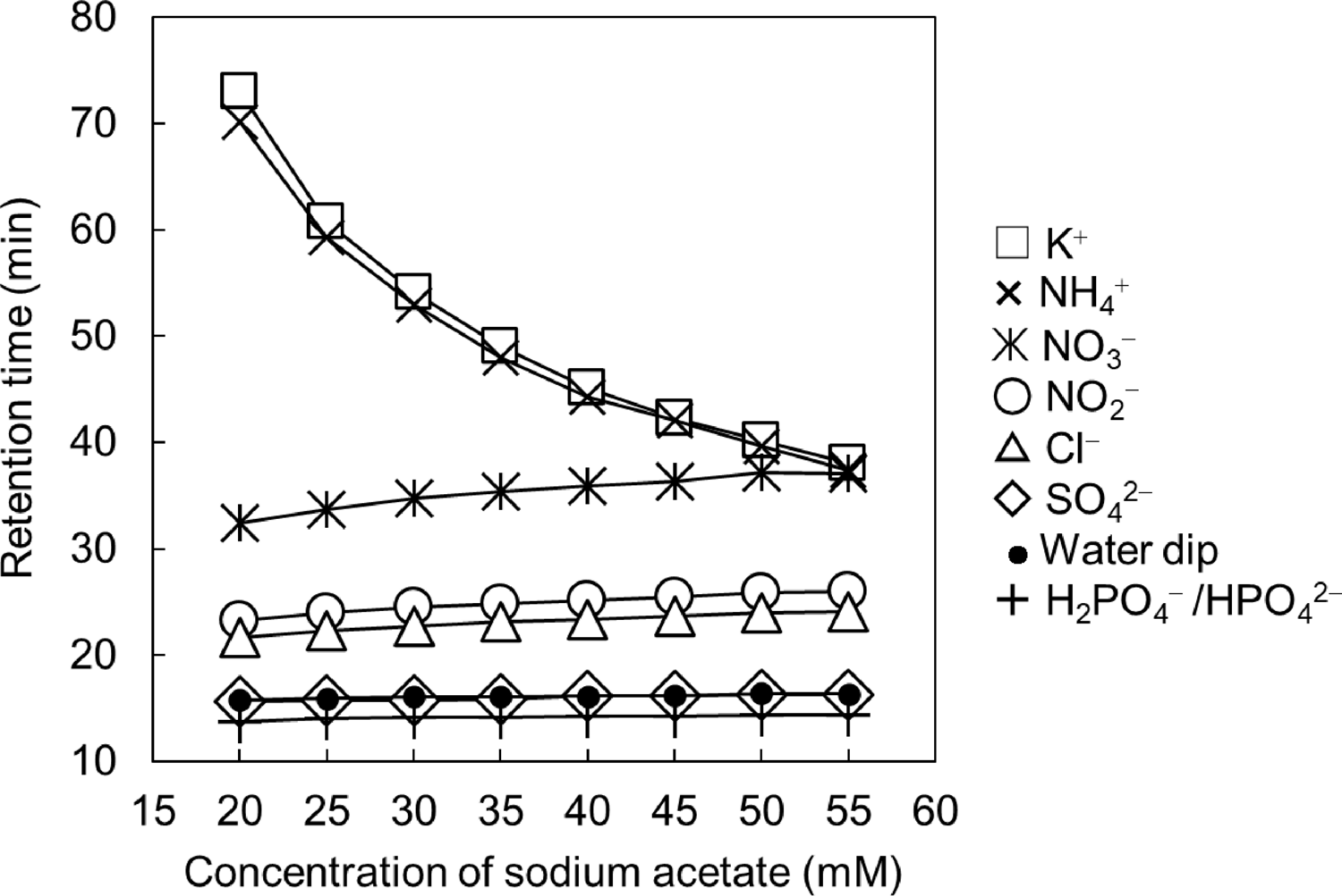
Effect of sodium acetate concentration on the retention time of the analyte ions. Conditions: Column, Three SeQuant ZIC-pHILIC column (150 mm × 4.6 mm i.d., particle size: 5 μm); column temperature, 40 °C; injection volume, 20 µL; eluent flow rate, 0.25 mL/min

**Fig. 2:**
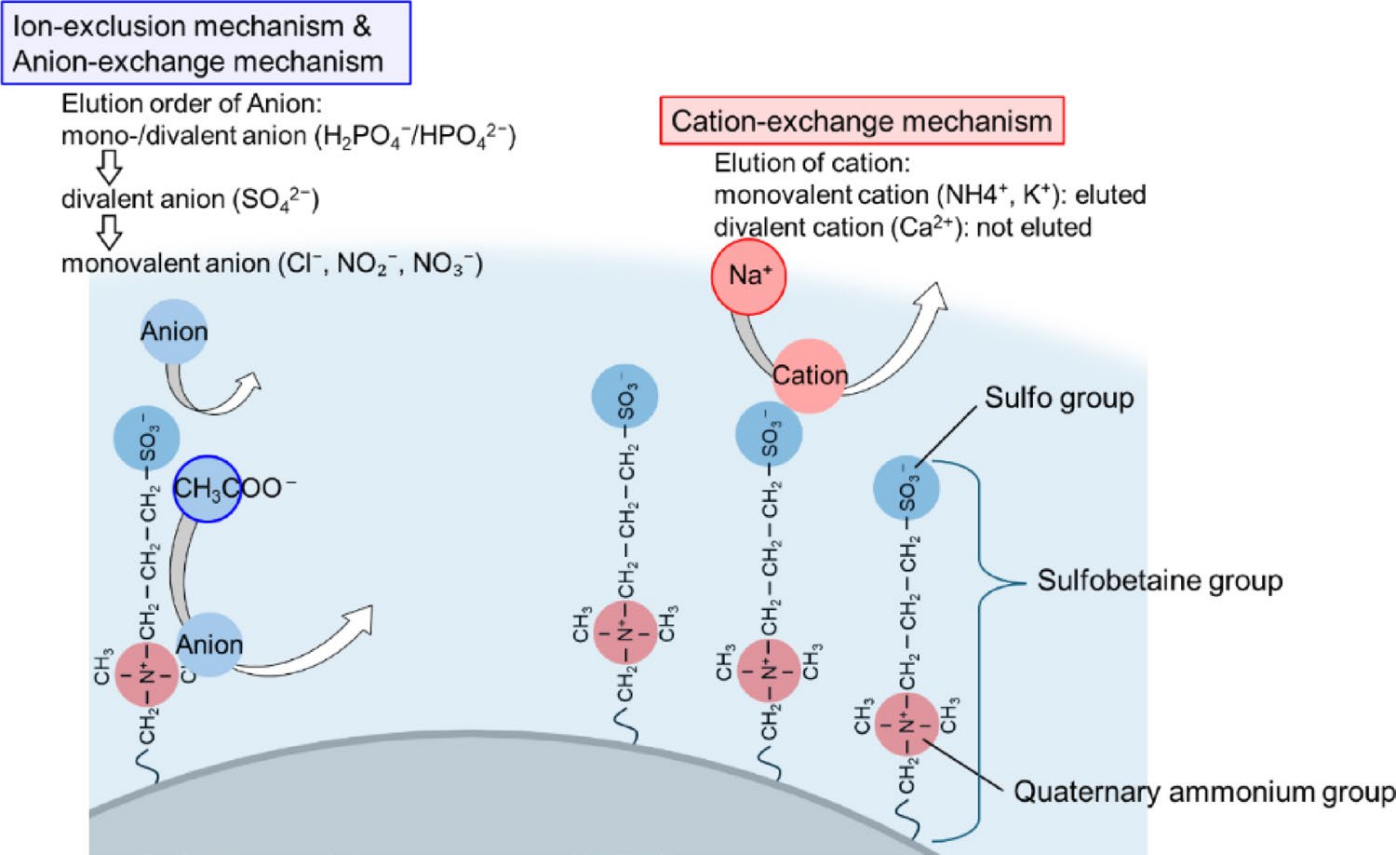
Schematic diagram of polymer-based zwitterionic exchanger, and expected separation mechanisms

The optimal concentration of sodium acetate in the eluent for obtaining simultaneous separation of analytes was investigated with the results shown in Fig. 1. The retention order was as follows: mono-/divalent anion (H_2_PO_4_^‒^/HPO_4_^2‒^) < divalent anion (SO_4_^2‒^) and eluent dip < monovalent anions (Cl^‒^, NO_2_^‒^, and NO_3_^‒^) < monovalent cations (K^+^ and NH_4_^+^) [5]. Based on the separation order, the ion-exclusion chromatographic (IEC) mechanism was expected to be the dominant separation mode for anions because H_2_PO_4_^‒^/HPO_4_^2‒^ and SO_4_^2‒^ were eluted faster than Cl^‒^, NO_2_^‒^, and NO_3_^‒^, as shown in Fig. 2. In addition, SO_4_^2‒^ was eluted after HPO_4_^2‒^/H_2_PO_4_^‒^, and this result indicated that the separation mechanism was not only limited to anion exclusion by a sulfo group, which was the dominant separation mode, but anion exchange by a quaternary ammonium group was also involved as the secondary separation mode, as shown in Fig. 2. Higher-resolution IEC separation for anions based on the sulfobetaine group compared with only the sulfo group was assumed to the IEC mechanism due to the terminal sulfo group and suppression of the exclusion force by the quaternary ammonium group on the stationary-phase side. By contrast, cations are separated based on the cation-exchange chromatographic (CEC) mechanism as the dominant separation mode because monovalent cations (K^+^, NH_4_^+^) were eluted from the separation column while divalent cation (Ca^2+^) were not eluted, as shown in Fig. 2 [5].

When the concentration of sodium acetate was increased from 20 to 55 mM, the cation retention time decreased from 73 to 38 min for K^+^ and from 70 to 37 min for NH_4_^+^, whereas the retention time of anions increased from 32 to 37 min for NO_3_^‒^, from 23 to 26 min for NO_2_^‒^, from 21 to 24 min for Cl^‒^, and from 13 to 14 min for H_2_PO_4_^‒^/HPO_4_^2‒^ as shown in Fig. 1. Based on the above results, although shorter retention times are preferable for higher analytical throughput, the peaks of NO_3_^‒^ and cations approached each other with increasing sodium acetate concentration, leading to the selection of 40 mM (theoretical pH: 8.2; measured pH: 7.71) as the optimal concentration. In addition, target analytes other than K^+^, and NH_4_^+^ were separated for this elution condition.

### Optimization of 18-crown-6 concentration in the eluent for separating NH_4_^+^ and K^+^

**Fig. 3:**
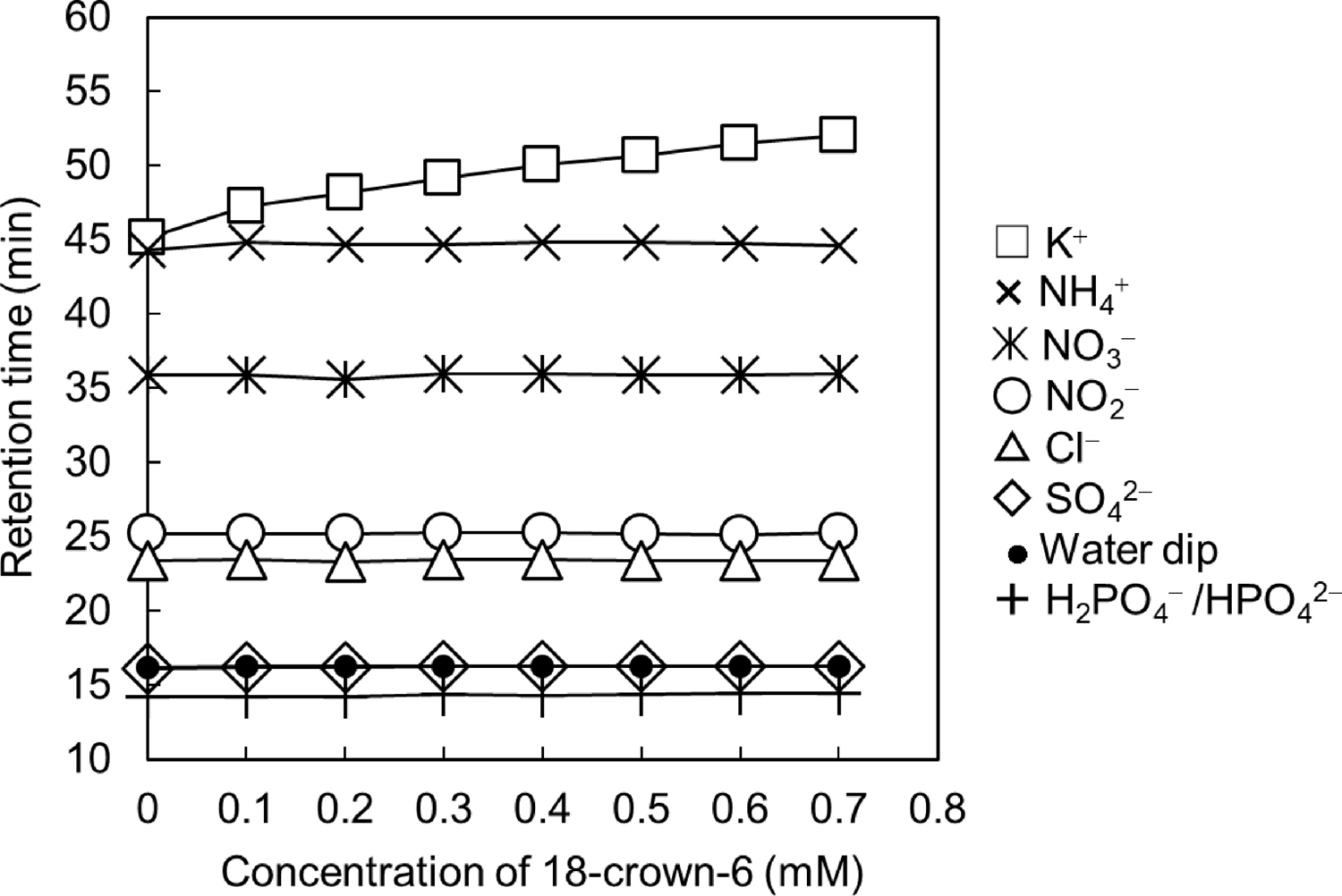
Effect of 18-crown-6 concentration on the retention time of the analyte ions. Conditions: Eluent: 40 mM sodium acetate. Other separation conditions were the same as those used to obtain the data presented in Fig. 1

**Fig. 4:**
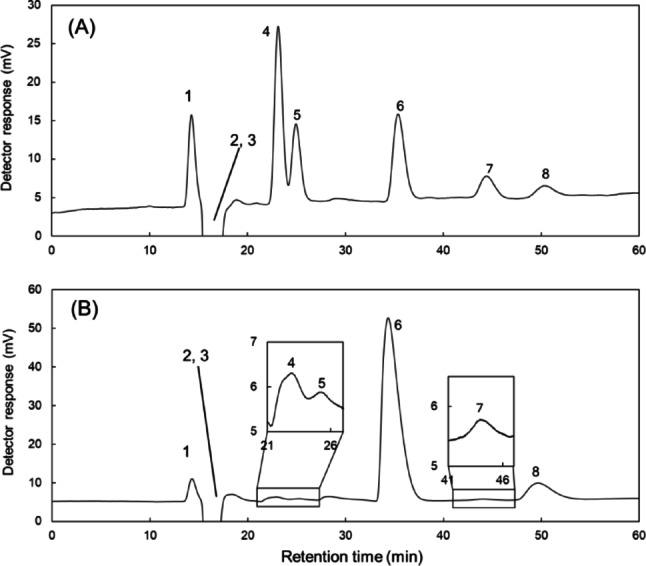
Chromatographic separation of target ionic nutrients in the (**A**) standard sample (3.0 mM KH_2_PO_4_, 3.0 mM NH_4_NO_3_, 2.0 mM NaNO_2_, 2.0 mM MgSO_4_, and 2.0 mM CaCl_2_) and (**B**) hydroponic solutions collected from hydroponic system on days 0 using the developed IEC/CEC method. Conditions: Eluent; 40 mM sodium acetate plus 0.5 mM 18-crown-6 (pH 7.71). Peak number: 1; H_2_PO_4_^‒^/HPO_4_^2‒^, 2; water dip, 3; SO_4_^2‒^, 4; Cl^‒^, 5; NO_2_^‒^, 6; NO_3_^‒^, 7; NH_4_^+^, and 8; K^+^.Other separation conditions were the same as those used to obtain the data presented in Fig. 3

As described in the previous section, when the sodium acetate concentration was increased from 20 to 55 mM, the peak resolution (*Rs*) between NH_4_^+^ and K^+^ was inadequate. Various concentrations of 18-crown-6 were added to the 40 mM sodium acetate eluent to improve the resolution between NH_4_^+^ and K^+^. The separation between NH_4_^+^ and K^+^ was based on the stability constant of the complexation of alkali metal ions with 18-crown-6 (log *K*_NH4+_ = 1.23, and log *K*_K+_  = 2.03) [26]. When included in the eluent, 18-crown-6 was absorbed onto the cation-exchange resin in the column, increasing the retention time of the associated monovalent cations. As shown in Fig. 3, the retention time of K^+^ increased with increasing 18-crown-6 concentration, leading to higher *Rs* so that *Rs* values of 0.692, 0.897, 1.153, 1.436, 1.593, 1.733, 2.176 were obtained for the 18-crown-6 concentrations of 0.1, 0.2, 0.3, 0.4, 0.5, 0.6, and 0.7 mM, respectively. The optimal concentration of 18-crown-6 was found to be 0.5 mM because Rs exceeded 1.5 for this concentration; additionally, for equal width peaks, the resolution of 1.5 accounted for an overlap of only 0.1%, which is considered sufficient for the baseline resolution of equal height peaks [27]. Based these results, simultaneous separation of all analyte ions was achieved using the developed IEC/CEC with 40 mM sodium acetate, and 0.5 mM 18-crown-6 (pH 7.71) as the eluent, as shown in Fig. 4A.

### Analytical performance

The separation performance of the target analytes under optimized eluent conditions is summarized in Table S1. For all ions, the calibration curves showed excellent linearity with correlation coefficients (*R*^2^) ranging from 0.9991 to 0.9999. The linear ranges were 0.025–1.0 mM for NO_2_^‒^, 0.05–1.0 mM for Cl^‒^, 0.25–30.0 mM for NO_3_^‒^, 0.25–5.0 mM for H_2_PO_4_^‒^/HPO_4_^2‒^, 0.5–4.0 mM for NH_4_^+^, and 0.5–30.0 mM for K^+^. The relative standard deviations (RSDs) of the peak areas and retention times determined from five consecutive injections of the standard solutions (3.0 mM KH_2_PO_4_, 3.0 mM NH_4_NO_3_, 2.0 mM NaNO_2_, and 2.0 mM CaCl_2_) ranged from 1.74 to 2.13% and from 0.19 to 1.28%, respectively. Recoveries obtained by the standard addition method (0.03 mL of 100 mM KH_2_PO_4_, NH_4_NO_3_, NaNO_2_, and CaCl_2_ added to 2.97 mL of day-0 liquid fertilizer) ranged from 99.0 to 101%. The limits of detection (LoD, S/N = 3.3) calculated based on the peak heights were 0.00820–0.0337 mM and 0.0993–0.150 mM for the anions and the cations, respectively, and the limits of quantification (LoQ, S/N = 10) were 0.0246–0.101 mM and 0.298–0.449 mM for the anions and the cations, respectively.

### Apply for the analysis of hydroponic fertilizer solution

**Fig. 5:**
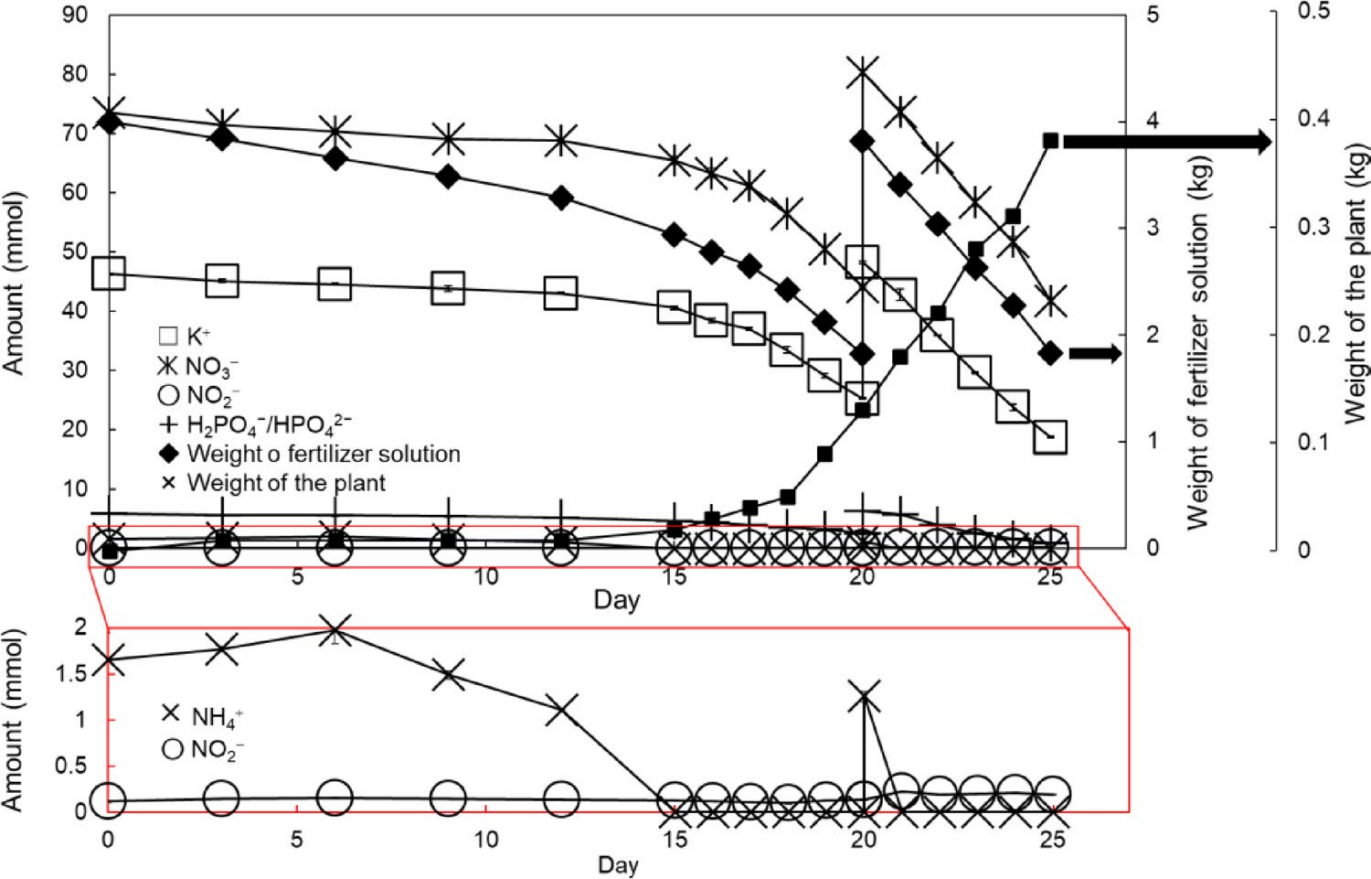
Temporal evolution of the amount (mmol) of inorganic ionic nutrients in the fertilizer solution, weight of the plant, and weight of the fertilizer solution during hydroponic cultivation

The IEC/CEC system was successfully applied to separate the ionic nutrients as shown in Fig. 4B. In addition, the temporal evolution of the concentration or amount of inorganic ionic nutrients in the fertilizer solution, weight of the plant, and weight of the fertilizer solution were monitored during the lettuce cultivation period in a closed hydroponic system using the data obtained by the developed IEC/CEC system, as shown in Fig. 5 and Fig. S2. The results showed that the weight of the liquid fertilizer gradually decreased from day 0 to 15, with an average depletion rate of 70.7 g/day, primarily due to evaporation and secondarily due to nutrient uptake by plants. For days 15–20, the average depletion rate increased to 224 g/day and was further accelerated to 398 g/day for days 20–25. The accelerated consumption of the nutrient solution corresponded to an enhanced plant growth rate (Fig. 5), indicating a shift in the major factor responsible for nutrient depletion from evaporation to plant uptake. Temporal evolution of the concentration (mmol/L) of inorganic ionic nutrients in the fertilizer solution are shown in Fig. S2 and Table S2. Among the major ionic nutrients, the concentration of H_2_PO_4_^‒^/HPO_4_^2‒^ remained nearly constant, whereas the concentration of K^+^ and NO_3_^‒^ increased with time. While ionic nutrient concentrations generally decrease due to plant uptake, they can also increase via concentration effects caused by water loss through plant growth and evaporation. Therefore, evaluation of the decreases in the concentration based solely on molarity can be misleading, in contrast to the calculation of the total amount (mmol) of major ionic species (NO_3_^‒^, K^+^, and H_2_PO_4_^‒^/HPO_4_^2‒^) which allowed accurate monitoring of their depletion trends. Therefore, analytical results in this study were expressed in terms of “amount (mmol)”. Amount of ionic nutrients decreased gradually during the first 15 days and declined sharply during days 15–25, corresponding to accelerated plant growth. The concentration of NO_2_^‒^ was low and essentially constant. Additionally, compared with major ionic nutrients, a lower amount of NH_4_^+^ was detected in the fertilizer solution. The amount of NH_4_^+^ dramatically decreased from day 12 to day 15 and after fertilizer addition (from day 20 to day 21). Nutrient solution volume showed a similar decreasing trend with accelerated plant growth. On day 20, when the liquid fertilizer volume dropped below 2.0 L, an additional 2.0 L of nutrient solution was added. As a result, the amounts of NO_3_^‒^, K^+^, and H_2_PO_4_^‒^/HPO_4_^2‒^ increased to 80.4, 48.3, and 6.37 mmol, respectively. At the end of cultivation, the concentration of H_2_PO_4_^‒^/HPO_4_^2‒^ decreased to 0.983 mmol, whereas NO_3_^‒^ (41.7 mmol) and K^+^ (18.7 mmol) concentrations remained relatively high due to the addition of the mixed nutrient solution. As discussed above, the concentration of ionic nutrients was comprehensively monitored using the developed IEC/CEC. All obtained data, including the data for the weight of the fertilizer solution, weight of the plant, and amount of analytes, are summarized in Table S3.

### Validation of accuracy in simultaneous analysis

**Fig. 6:**
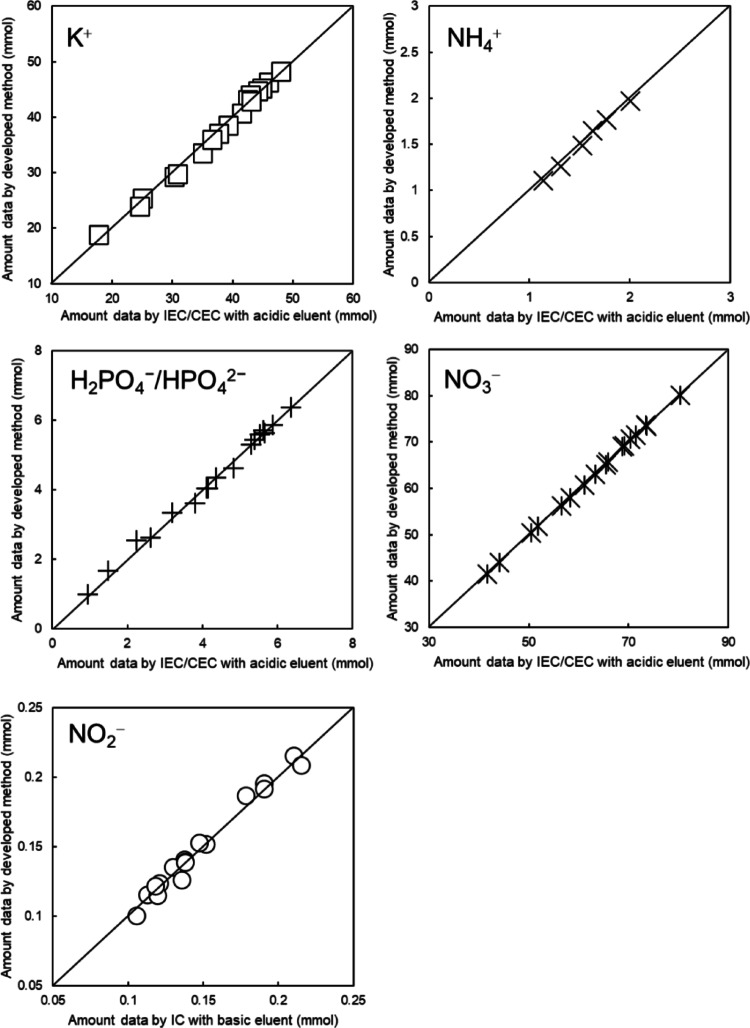
Comparison of analyte ion concentrations obtained using the developed IEC/CEC method with neutral eluent to those obtained using the IEC/CEC method with acidic eluent and IC with basic eluent

In a proof-of-concept demonstration of the application of the IEC/CEC method to hydroponics, H_2_PO_4_^‒^/HPO_4_^2‒^, NO_3_^‒^, NO_2_^‒^, and K^+^ levels in a hydroponic fertilizer solution were monitored. To evaluate the accuracy of the developed method, the concentrations of H_2_PO_4_^‒^/HPO_4_^2‒^, NO_3_^‒^, and K^+^ obtained by the developed IEC/CEC method were compared with the results obtained by the previously established IEC/CEC method with acidic eluent. In addition, the concentrations of NO_2_^‒^ obtained by the developed IEC/CEC method were compared with those obtained using the IC method with basic eluent. High correlations were observed between the concentrations obtained by the developed method and the concentrations obtained using the previously established methods, as shown in Fig. 6. These results demonstrate that the proposed method provides high analytical accuracy and is suitable for monitoring ionic nutrients in hydroponic fertilizer solutions.

## Conclusion

In this study, IEC/CEC with neutral eluent (40 mM sodium acetate, and 0.5 mM 18-crown-6, pH 7.71) was developed for simultaneous monitoring of ionic nutrients (NO_3_^‒^, NO_2_^‒^, NH_4_^+^, H_2_PO_4_^‒^/HPO_4_^2‒^, K^+^) as an alternative to the use of pH and EC measurements. The use of neutral eluent and polymer-based stationary phase chemically bonded through zwitterionic functional groups avoided the degradation of the separation column by the precipitation of metal species that occurs under alkaline elution conditions as well as the oxidation of NO_2_^‒^ to NO_3_^‒^ under an acidic eluent and the instability of separation quality observed in the absence of the substitution of the aqueous neutral eluent by organic solvent such as acetonitrile after each use. The developed IEC/CEC was successfully applied to monitor the temporal evolution of the amount of target ionic nutrients in hydroponic solutions. Using the IEC/CEC method, the target ionic nutrients were comprehensively monitored, and their consumption during hydroponic plant growth was demonstrated using the obtained ion concentration data. Future work will focus on examining the feasibility of the implementation of this technique for real-time monitoring and control of nutrient solutions in small- to medium-scale hydroponic facilities.

## Supplementary Information

Below is the link to the electronic supplementary material.

Supplementary Material 1

## Data Availability

The data underlying this article are available in the article and in its online supplementary material.
