# Supplementary material for: Development of the simultaneous determination of anionic and cationic nutrients in hydroponic solutions using polymer-based zwitterionic exchanger with neutral eluent
Source: Anal Sci. 2026 May 19;42(8):735–43. doi: 10.1007/s44211-026-00922-0 (PMC13400577; doi:10.1007/s44211-026-00922-0)
Supplement: Supplementary file 1 — Supplementary Material 1 [file 44211_2026_922_MOESM1_ESM.docx]

**Supplementary Material**

**Development of the simultaneous determination of anionic and cationic nutrients in** **hydroponic solutions using polymer-based zwitterionic exchanger with neutral eluent**

Yuki Uga ^1^, Atsushi Hashigami ^1^, Haruki Tsuboi ^1^, Yamato Okada ^1^, Yuta Mitsui ^1^, Taku Fujiwara ^2^, Yuki Sago ^3^, Daisuke Kozaki ^1†^

^1^ Department of Chemistry and Biotechnology, Faculty of Science and Technology, Kochi University, 2-5-1 Akebono-cho, Kochi city, Kochi 780-8520, Japan

^2^ Department of Environmental Engineering, Kyoto University, C1-222, Nishikyo-ku, Kyoto 615-8540, Japan

^3^ Graduate School of Sciences and Technology for Innovation, Yamaguchi University, Yoshida 1677-1, Yamaguchi 753-8515, Japan

^†^ Corresponding author.

E-mail: daisuke.2-10@kochi-u.ac.jp

**Contents**

Table S1. Analytical performance under optimal conditions. 2

Table S2. Data of the temporal evolution of the concentration (mmol/L) of inorganic ionic nutrients in the fertilizer solution, weight of the plant, and weight of the fertilizer solution during hydroponic cultivation. 3

Table S3. Data of the temporal evolution of the amount (mmol) of inorganic ionic nutrients in the fertilizer solution, weight of the plant, and weight of the fertilizer solution during hydroponic cultivation. 4

Figure S1. Hydroponic cultivation system used in this study. 5

Figure S2. Temporal evolution of the concentration (mmol/L) of inorganic ionic nutrients in the fertilizer solution, weight of the plant, and weight of the fertilizer solution during hydroponic cultivation. 6

Table S1 Analytical performance under optimal conditions.

| Analyte | Linearity range,  mmol/L | Correlation coefficient, *R*^2^ | ^*^RSD, % (*n* = 5) | | ^**^LoD,  mmol/L (S/N = 3.3) | ^***^LoQ,  mmol/L (S/N = 10) | Additional recovery, %  (*n* = 3) |
| --- | --- | --- | --- | --- | --- | --- | --- |
|  |  |  | Peak area | Retention time |  |  |  |
| K^+^ | 0.50–30 | 0.9999 | 2.03 | 0.19 | 0.150 | 0.449 | 99.0 ± 0.742 |
| NH_4_^+^ | 0.5–4.0 | 0.9998 | 2.13 | 0.33 | 0.0993 | 0.298 | 99.3 ± 1.77 |
| NO_2_^－^ | 0.025–1.0 | 0.9999 | 1.93 | 0.30 | 0.00820 | 0.0246 | 100 ± 0.366 |
| NO_3_^－^ | 0.25–30 | 0.9999 | 1.81 | 0.45 | 0.0167 | 0.0501 | 100 ± 1.18 |
| H_2_PO_4_^－^/HPO_4_^2－^ | 0.25–5.0 | 0.9993 | 1.81 | 1.28 | 0.0337 | 0.101 | 101 ± 1.10 |
| Cl^－^ | 0.050–1.0 | 0.9999 | 1.74 | 0.48 | 0.0156 | 0.0469 | 99.4 ± 0.413 |

*RSD, relative standard deviation; **LoD, limit of detection; ***LoQ, limit of quantification

Table S2. Data of the temporal evolution of the concentration (mmol/L) of inorganic ionic nutrients in the fertilizer solution, weight of the plant, and weight of the fertilizer solution during hydroponic cultivation.

|  | | Date of sampling | | | | | | | | | | | | | | | | | |
| --- | --- | --- | --- | --- | --- | --- | --- | --- | --- | --- | --- | --- | --- | --- | --- | --- | --- | --- | --- |
|  |  | 0 | 3 | 6 | 9 | 12 | 15 | 16 | 17 | 18 | 19 | 20 | 20 | 21 | 22 | 23 | 24 | 25 |  |
| Weight of fertilizer solution (kg) | | 4.00 | 3.84 | 3.66 | 3.49 | 3.29 | 2.94 | 2.78 | 2.65 | 2.42 | 2.12 | 1.82 | 3.82 | 3.41 | 3.04 | 2.63 | 2.28 | 1.83 |  |
| Weight of plant (kg) | | 0.00 | 0.01 | 0.01 | 0.01 | 0.01 | 0.02 | 0.03 | 0.04 | 0.05 | 0.09 | 0.13 | 0.13 | 0.18 | 0.22 | 0.28 | 0.31 | 0.38 |  |
| NO_3_^－^ | (mmol/L) | 18.4 | 18.6 | 19.2 | 19.8 | 20.9 | 22.3 | 22.8 | 23.1 | 23.4 | 23.8 | 24.2 | 21.0 | 21.6 | 21.7 | 22.2 | 22.7 | 22.8 |  |
| NO_2_^－^ |  | 0.0293 | 0.0385 | 0.0426 | 0.0414 | 0.0409 | 0.0441 | 0.0431 | 0.0428 | 0.0401 | 0.0596 | 0.0743 | 0.0352 | 0.0648 | 0.0625 | 0.0750 | 0.0914 | 0.104 |  |
| H_2_PO_4_⁻/HPO_4_^2^⁻ |  | 1.47 | 1.47 | 1.53 | 1.56 | 1.61 | 1.58 | 1.57 | 1.52 | 1.49 | 1.57 | 1.40 | 1.67 | 1.68 | 1.33 | 0.997 | 0.735 | 0.537 |  |
| NH_4_⁺ |  | 0.412 | 0.560 | 0.538 | 0.426 | 0.336 | 0.00 (N.D.) | 0.00 (N.D.) | 0.00 (N.D.) | 0.00 (N.D.) | 0.00 (N.D.) | 0.00 (N.D.) | 0.330 | 0.00 (N.D.) | 0.00 (N.D.) | 0.00 (N.D.) | 0.00 (N.D.) | 0.00 (N.D.) |  |
| K⁺ |  | 11.6 | 11.7 | 12.2 | 12.6 | 13.1 | 13.8 | 13.8 | 14.0 | 13.8 | 13.8 | 13.9 | 12.6 | 12.5 | 11.8 | 11.3 | 10.4 | 10.2 |  |

*N.D.: Not detected

Table S3. Data of the temporal evolution of the amount (mmol) of inorganic ionic nutrients in the fertilizer solution, weight of the plant, and weight of the fertilizer solution during hydroponic cultivation.

|  | | Date of sampling | | | | | | | | | | | | | | | | | |
| --- | --- | --- | --- | --- | --- | --- | --- | --- | --- | --- | --- | --- | --- | --- | --- | --- | --- | --- | --- |
|  |  | 0 | 3 | 6 | 9 | 12 | 15 | 16 | 17 | 18 | 19 | 20 | 20 | 21 | 22 | 23 | 24 | 25 |  |
| Weight of fertilizer solution (kg) | | 4.00 | 3.84 | 3.66 | 3.49 | 3.29 | 2.94 | 2.78 | 2.65 | 2.42 | 2.12 | 1.82 | 3.82 | 3.41 | 3.04 | 2.63 | 2.28 | 1.83 |  |
| Weight of plant (kg) | | 0.00 | 0.01 | 0.01 | 0.01 | 0.01 | 0.02 | 0.03 | 0.04 | 0.05 | 0.09 | 0.13 | 0.13 | 0.18 | 0.22 | 0.28 | 0.31 | 0.38 |  |
| NO_3_^－^ | (mmol) | 73.5 | 71.4 | 70.3 | 69.1 | 68.8 | 65.5 | 63.3 | 61.1 | 56.5 | 50.5 | 44.1 | 80.4 | 73.6 | 65.9 | 58.3 | 51.8 | 41.7 |  |
| NO_2_^－^ |  | 0.117 | 0.147 | 0.156 | 0.144 | 0.135 | 0.130 | 0.120 | 0.113 | 0.0970 | 0.126 | 0.135 | 0.134 | 0.221 | 0.190 | 0.197 | 0.208 | 0.191 |  |
| H_2_PO_4_⁻/HPO_4_^2^⁻ |  | 5.88 | 5.63 | 5.58 | 5.44 | 5.29 | 4.63 | 4.35 | 4.03 | 3.61 | 3.34 | 2.55 | 6.37 | 5.72 | 4.03 | 2.62 | 1.68 | 0.983 |  |
| NH_4_⁺ |  | 1.65 | 1.77 | 1.97 | 1.49 | 1.11 | 0.00 (N.D.) | 0.00 (N.D.) | 0.00 (N.D.) | 0.00 (N.D.) | 0.00 (N.D.) | 0.00 (N.D.) | 1.26 | 0.00 (N.D.) | 0.00 (N.D.) | 0.00 (N.D.) | 0.00 (N.D.) | 0.00 (N.D.) |  |
| K⁺ |  | 46.3 | 45.1 | 44.6 | 43.9 | 43.0 | 40.6 | 38.4 | 37.0 | 33.5 | 29.2 | 25.3 | 48.3 | 42.7 | 35.9 | 29.6 | 23.8 | 18.7 |  |

*N.D.: Not detected


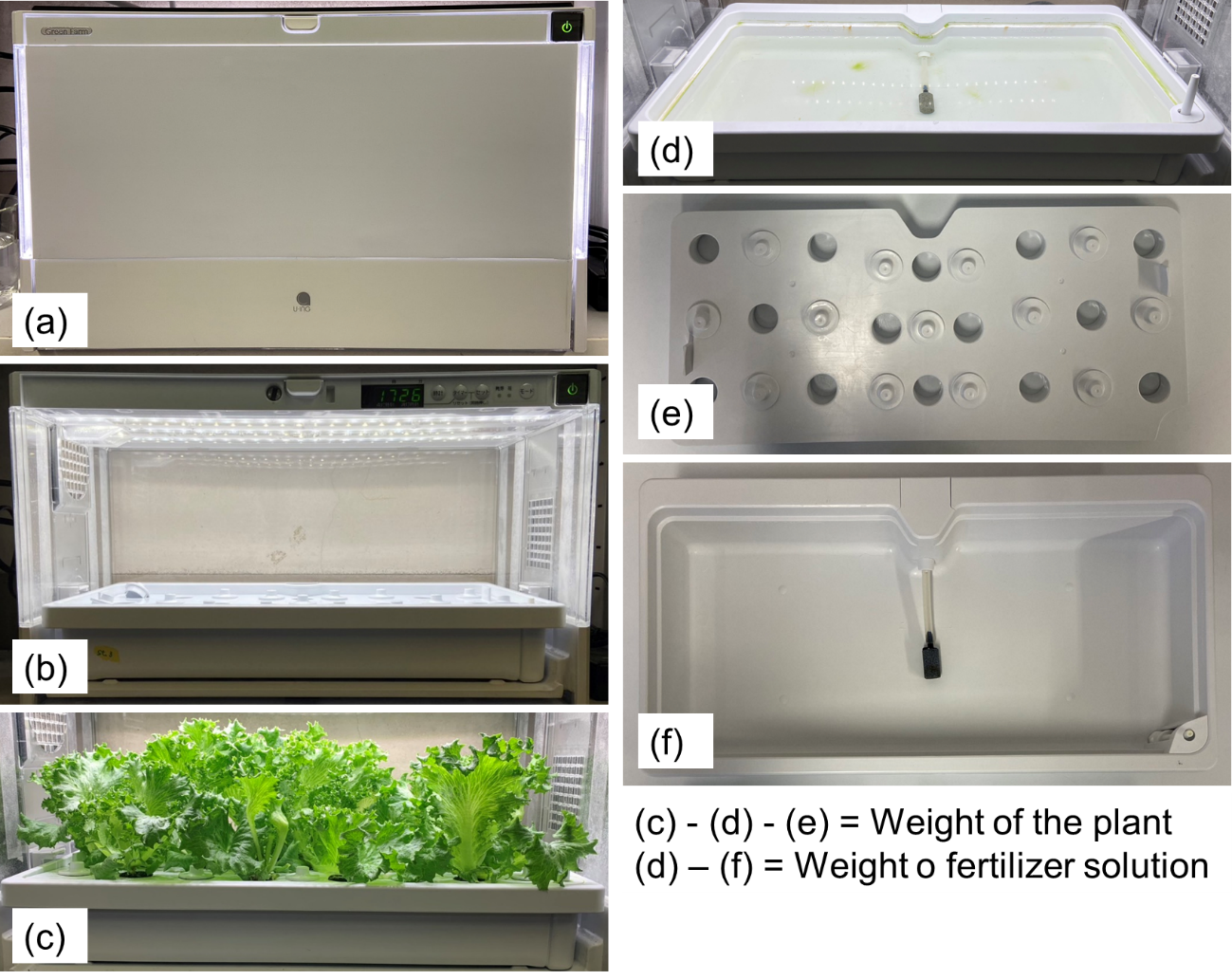


Figure S1 Hydroponic cultivation system used in this study. Photos of (a) the entire system, (b) seeding panel and fertilizer solution storage, (c) seeding panel, lettuce, fertilizer solution storage, and fertilizer solution, (d) fertilizer solution storage and fertilizer solution, (e) seeding panel, and (f) fertilizer solution storage.


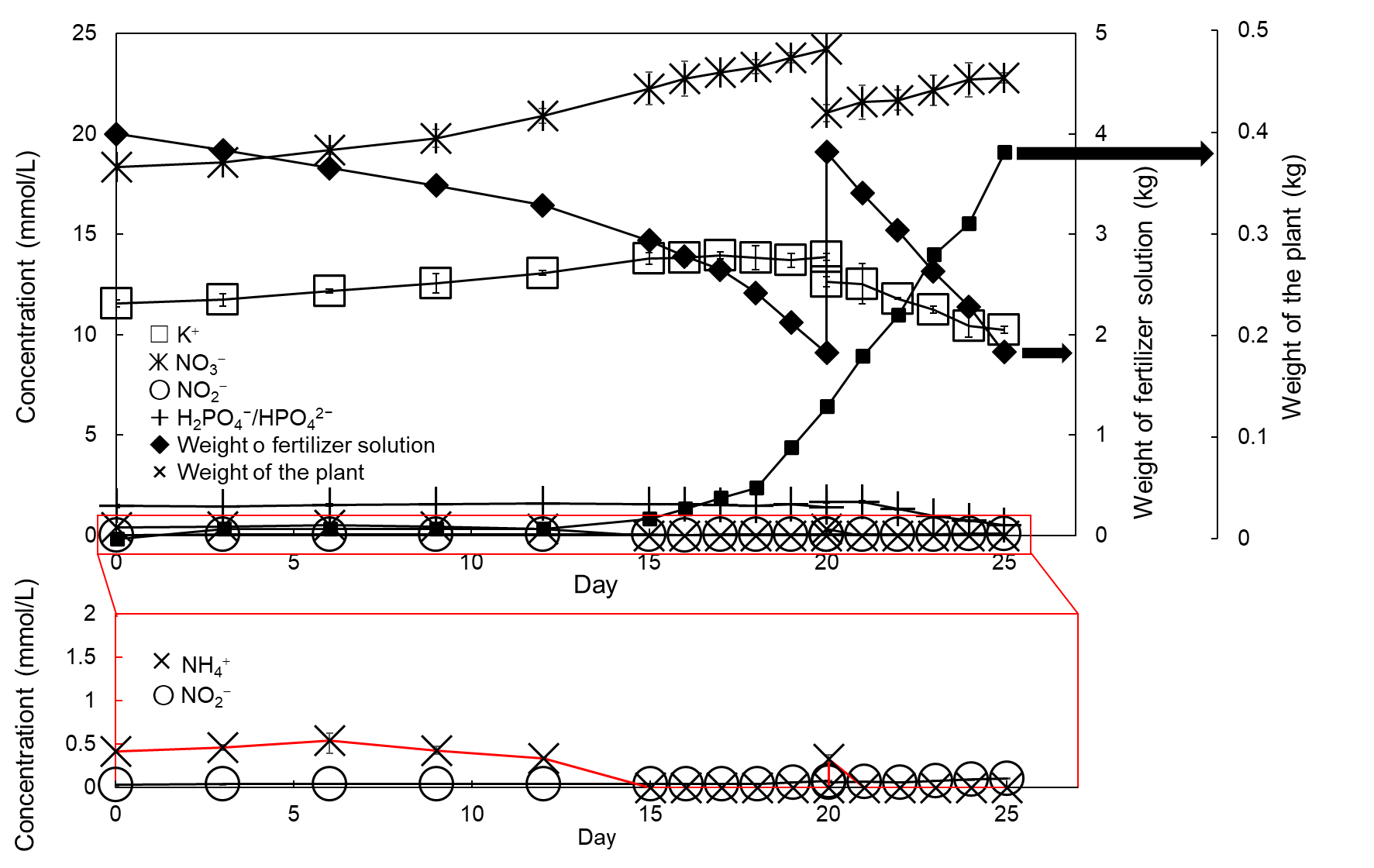


Figure S2 Temporal evolution of the concentration (mmol/L) of inorganic ionic nutrients in the fertilizer solution, weight of the plant, and weight of the fertilizer solution during hydroponic cultivation.
